# Differential sensitivity of Glioma stem cells to Aurora kinase A inhibitors: Implications for stem cell mitosis and centrosome dynamics

**DOI:** 10.1016/j.scr.2014.05.001

**Published:** 2014-07

**Authors:** Mariella Mannino, Natividad Gomez-Roman, Helfrid Hochegger, Anthony J. Chalmers

**Affiliations:** a Genome Damage and Stability Centre, University of Sussex, Brighton BN19RQ, UK; b Institute of Cancer Sciences, University of Glasgow, Glasgow G12 8QQ, UK

## Abstract

Glioma stem-cell-like cells are considered to be responsible for treatment resistance and tumour recurrence following chemo-radiation in glioblastoma patients, but specific targets by which to kill the cancer stem cell population remain elusive. A characteristic feature of stem cells is their ability to undergo both symmetric and asymmetric cell divisions. In this study we have analysed specific features of glioma stem cell mitosis. We found that glioma stem cells appear to be highly prone to undergo aberrant cell division and polyploidization. Moreover, we discovered a pronounced change in the dynamic of mitotic centrosome maturation in these cells. Accordingly, glioma stem cell survival appeared to be strongly dependent on Aurora A activity. Unlike differentiated cells, glioma stem cells responded to moderate Aurora A inhibition with spindle defects, polyploidization and a dramatic increase in cellular senescence, and were selectively sensitive to Aurora A and Plk1 inhibitor treatment. Our study proposes inhibition of centrosomal kinases as a novel strategy to selectively target glioma stem cells.

## Highlights

• Glioma stem cells (GSC) exhibit aberrant cell division and increased polyploidy
• Centrosome maturation is abnormal and GSC survival is Aurora A dependent
• Inhibition of Aurora A increases spindle defects, polyploidy and senescence in GSC
• GSC are selectively sensitive to Aurora A and Plk1 inhibitors
• This is a novel strategy to target GSC and improve outcomes in glioblastoma

## Introduction

In the past decade, stem-cell-like cancer cells have been identified in several tumours and implicated in treatment resistance. Glioblastoma is one of the most extensively studied cancer types in relation to treatment resistance and the cancer stem cell (CSC) model. This is probably due to the poor outcome of patients treated for this disease (median overall survival of 14.6 months) (Stupp et al., 2009) and to the almost inevitable recurrence following chemo-radiation, which renders glioblastomas a valuable model for study of cancer cell resistance to radiation and chemotherapy. Several clinical series have found a correlation between glioma stem cell (GSC) features in patient specimens (expression of putative GSC markers, neurosphere formation ability *in vitro*) and tumour recurrence and poorer prognosis (Strojnik et al., 2007; Zhang et al., 2008; Zeppernick et al., 2008; Pallini et al., 2008). Furthermore, a recent study using a genetically engineered mouse model of glioma identified a relatively quiescent subpopulation of cells that was responsible for post-chemotherapy tumour growth, through its capacity to produce transient subsets of highly proliferating cells (Chen et al., 2012). These findings reinforced the rationale for the GSC theory and highlighted the importance of processes regulating cellular growth, such as DNA replication and mitosis, in the context of treatment resistance.

Current standard therapy for glioblastoma consists of debulking surgery followed by radiation with concomitant and adjuvant administration of the alkylating agent temozolomide (Stupp et al., 2009). Most studies investigating intrinsic GSC resistance have focused on the role of DNA damage responses and repair. However, experimental data from these studies are conflicting and response mechanisms of GSCs to radiation and chemotherapy remain controversial (Beier et al., 2011). Other stem cell features, such as those involved in regulating cell division, are only poorly understood in GSCs, but could provide clinically relevant targets to improve treatment outcomes. A paramount feature of stem cell mitosis is the ability to divide both symmetrically and asymmetrically, giving rise to differentiating daughter cells as well as maintaining a pool of self-renewing stem cells (Lathia et al., 2011). Studies in fruit fly neuronal stem cells have demonstrated a critical role for the centrosome in establishing asymmetry during mitosis (Yamashita et al., 2007). Centrosomes are the microtubule organising centres in animal cells that form the poles of the mitotic spindle. They are regulated by several mitotic kinases including Aurora kinase A (AurA), Polo-like kinase 1 (Plk1) and Cyclin dependent kinase 1 (Cdk1) (Nigg and Stearns, 2011). Inhibitors of these kinases have long been implicated as potential cancer therapies (Harrison et al., 2009). A recent study has highlighted Plk1 inhibition as a strategy to selectively kill GSC enriched populations (Lee et al., 2012). Moreover, AurA is overexpressed in glioblastomas (Loh et al., 2010; Lehman et al., 2012); its expression levels have been correlated with patient outcome (Barton et al., 2010); and data from other tumour sites suggest a role for AurA in CSC behaviour (Cammareri et al., 2010; Chefetz et al., 2011). However, a link between centrosome biology, mitotic kinase inhibition and GSC targeting has not been established.

In this study we analysed centrosome and mitotic spindle morphology in glioblastoma stem cell enriched and differentiated populations. We report a higher frequency of abnormal spindles and a more pronounced maturation of centrosomes preceding mitosis in GSC enriched populations. This prompted us to investigate whether differences in mitotic spindle dynamics could provide a novel therapeutic strategy by which GSC populations could be specifically targeted. We show that AurA and Plk1, both involved in centrosome maturation and bipolar spindle assembly can be targeted to kill GSCs more effectively, and propose that this strategy might improve outcomes for patients with glioblastoma.

## Results

### Neurosphere cultures of primary glioblastoma cells generate invasive intracranial xenografts in immunodeficient mice

**Figure 1:**
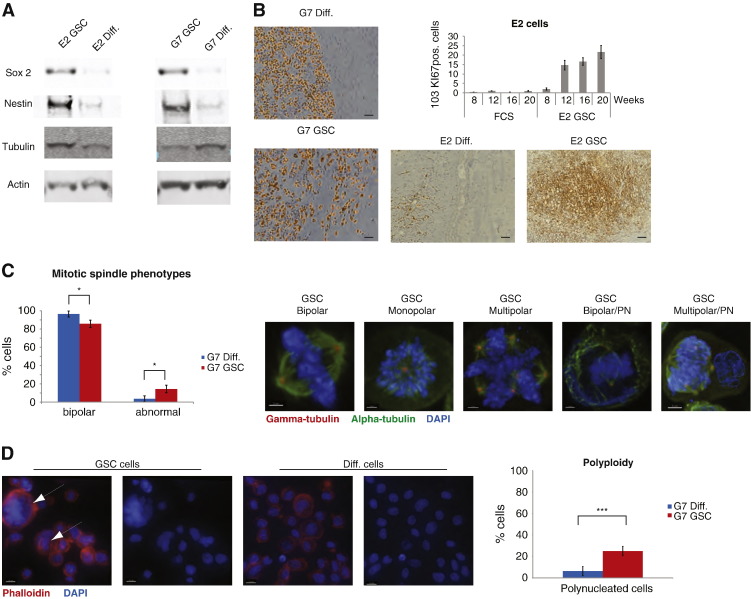
Primary glioblastoma cells cultured as neurospheres in serum-free conditions express high levels of stem cell markers, form invasive intracranial xenografts in immunodeficient mice and have a higher frequency of abnormal mitotic spindles and a distinct pattern of centrosome maturation. (A) Immunoblot analysis of lysates of E2 and G7 primary glioblastoma cell cultures was performed, probing for the stem cell markers Sox2 and nestin, with tubulin and actin as loading controls (GSCs—glioma stem cells cultured as neurospheres in serum-free medium, diff.—differentiated, adherent cells cultured in serum-containing medium) (B) Immunohistochemistry was performed on sections of brains of CD1 nude mice that had been injected with 10^5^ E2 or G7 glioblastoma cells that had been cultured to enrich for GSC or to promote differentiation. Sections were stained for Ki67 (G7 images on left) to interrogate local brain invasion and for HLA-1ABC to identify tumour cells of human origin (E2 images below histogram). To measure tumour cell burden in highly infiltrative E2 derived xenografts, quantitative analysis of Ki67 positive cells in whole brain sections was performed using ZenBlue software. Number of Ki67 positive cells per section was plotted against time after injection of E2 GSC and differentiated cells (Scale bar at 50 μm). (C) Cells were stained for α tubulin (green), γ tubulin (red) and DAPI (blue), to visualise the mitotic spindle morphology: on the left, a diagram shows the percentages of normal and abnormal spindles in glioma stem cells and differentiated cells. On the right are representative images of mitotic phenotypes (Scale bar 2 μm). PN: polynucleated cells. An average of 26 mitotic cells/condition/experiment were identified randomly and scored (*p = 0.0232, two tailed *t*-test). (D) Cells were stained with phalloidin (red) and DAPI (blue) to visualise the cell cortex and nucleus: on the left are representative images of GSC and diff. cells (scale bar 10 μm); on the right, a diagram shows the percentages of polynucleated cells in the two populations. An average of 251 cells/condition/experiment were identified randomly and scored (***p = 0.000275, two tailed *t*-test).

Consistent with previously published data (Fael Al-Mayhani et al., 2009), primary glioblastoma cells cultured as non-adherent neurospheres in serum-free medium expressed high levels of stem cell markers including CD133, nestin and Sox2 (Fig. 1A, supplementary Fig. S1A,B), low levels of astrocytic differentiation markers including GFAP (supplementary Fig. S1B) and generated invasive intracranial xenografts in CD1 nude mice. Intracranial injection of 10^5^ E2 cells cultured as neurospheres (‘E2 GSC’) generates tumours in 100% of mice and these tumours were highly invasive. In addition to a mass at the injection site (Fig. 1B), tumour cells identified by human specific HLA-1ABC and Ki67 staining were detected throughout both hemispheres (Supplementary Fig. S1C). Quantitative analysis of Ki67 positive cells in whole brain slices harvested at various timepoints demonstrated increasing tumour cell burden up to 20 weeks after injection (Fig. 1B, supplementary Fig. S1C). In contrast, injection of 10^5^ E2 cells cultured as monolayers in serum-containing medium (‘E2 diff’) failed to generate tumour masses and tumour cells did not infiltrate the brain. Very low numbers of HLA-1ABC or Ki67 positive cells were detected (Fig. 1B). Injection of 10^5^ G7 cells cultured as neurospheres (‘G7 GSC’) generated tumours in 100% of mice; all tumours were highly invasive at the tumour margins (Fig. 1B). Injection of 10^5^ G7 cells cultured as monolayers in serum-containing medium (‘G7 diff’) also generated tumours but these had well defined edges and did not exhibit the invasive pattern observed in G7 GSC derived tumours (Fig. 1B).

### Glioblastoma stem cells are prone to mitotic failure and show a distinct pattern of centrosome maturation

Cell division of GSCs remains scarcely characterised, despite its role in maintaining stemness and generating cellular diversity (Lathia et al., 2011). To understand whether mitosis in these cells presents specific features that can be targeted therapeutically, we analysed their mitotic spindles by immuno-fluorescence. GSC enriched populations had a significantly higher frequency of abnormal mitotic spindles (monopolar or multipolar) compared to more differentiated populations: 14% *vs.* 4%, respectively (Fig. 1C). While scoring mitosis in the GSC enriched populations we frequently observed cells with two or more nuclei (Fig. 1C). To clarify whether these were cell aggregates or truly polyploid cells, we stained both cell populations with phalloidin to visualise the cell cortex. This allowed us to differentiate between single cells with two or more nuclei and closely attached cells with two single nuclei. Consistent with the mitotic spindle data, this analysis revealed that GSC enriched populations had a much higher percentage of polyploid cells compared to more differentiated populations: 25% *vs.* 6%, respectively (Fig. 1D). In order to test whether the increase in abnormal spindles was due to growth in suspension, we analysed spindle phenotypes in differentiated cells cultured as non-adherent aggregates and found that all imaged cells had bipolar spindles (data not shown), suggesting that the neurosphere growth is not a confounding factor for the observed mitotic phenotypes. To our knowledge, this is the first study reporting a higher frequency of abnormal mitotic spindles and polyploidy in GSC enriched populations *in vitro*.

**Figure 2:**
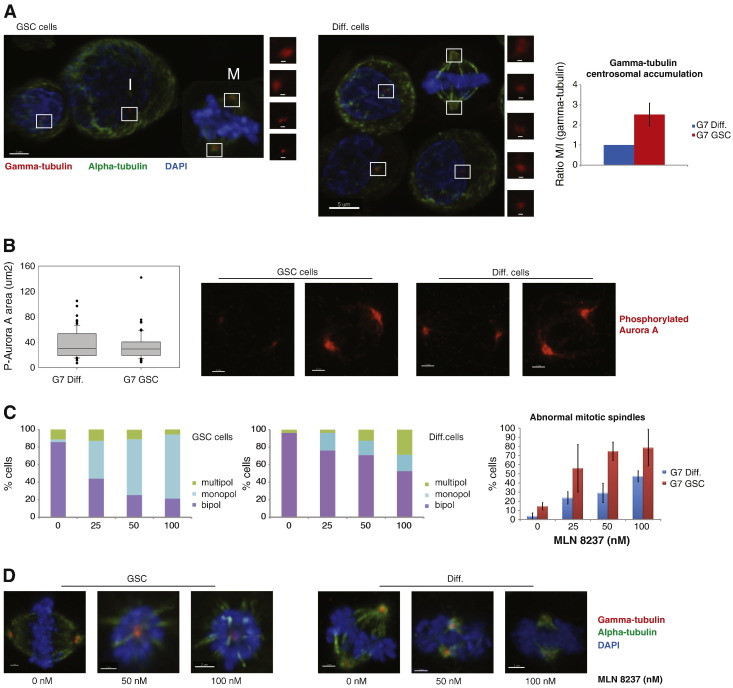
Glioblastoma stem cells have more abnormal spindles following treatment with Aurora A inhibitors. (A) Cells were stained for α tubulin (green), γ tubulin (red) and DAPI (blue), to visualise the accumulation of γ tubulin during centrosome maturation: at the top are representative images of GSC and diff. cells (scale bar 5 μm), with inserts showing γ tubulin staining in interphase (I) and mitotic (M) cells (scale bar 500 nm); at the bottom, a diagram shows the ratio between centrosomal γ-tubulin in mitotic and interphase cells, as a measure of centrosome maturation, in GSC and diff. populations. An average of 144 cells/condition/experiment were identified randomly and scored. All results are representative of three independent experiments. Error bars indicate means ± SD. (B) Cells were stained for P-AurA (red) and mitotic cells were analysed: on the left, a box plot shows the quantification of P-AurA in mitotic centrosomes in diff. and GSC cells; on the right are representative images of mitotic cells in the two populations (scale bar 2 μm). An average of 22 mitotic cells/condition/experiment were identified randomly and scored. (C–D) Cells were treated with MLN8237 (0, 25, 50 and 100 nM) and after 24 h they were fixed and stained for α tubulin (green), γ tubulin (red) and DAPI (blue), to visualise the mitotic spindle morphology. (B) On the left, two diagrams show the distribution of different spindle phenotypes in GSC and diff. cells; on the right a diagram shows the percentages of abnormal mitotic spindles in the two populations. (C) Representative images of GSC and diff. cells ± MLN8237 (scale bar 2 μm). An average of 26 mitotic cells/condition/experiment were identified randomly and scored. All results are representative of three independent experiments. Error bars indicate means ± SD.

While analysing spindle morphology we noted a distinct pattern in the distribution of γ-tubulin at the centrosomes in the GSC populations that was characterised by a marked increase in γ-tubulin at the mitotic centrosomes compared to differentiated cells. Given the role of centrosomes in bipolar spindle assembly, we further analysed the distribution of γ-tubulin in the two glioblastoma cell populations. For this purpose we measured the size of the γ-tubulin area in both interphase and mitotic cells (Fig. 2A). The specificity of the centrosomal γ-tubulin staining was confirmed by co-localization with centrin (supplementary Fig. S2A). This quantitative analysis revealed a more pronounced centrosomal accumulation of γ-tubulin at mitosis in GSCs, compared to differentiated cells (Fig. 2A). Thus, centrosome maturation, measured as the ratio between centrosomal γ-tubulin in mitotic and interphase cells, was increased more than twofold in GSCs. This finding, together with the difference in mitotic phenotypes, highlights the importance of investigating in detail GSC division and the processes leading to it, in order to find effective and specific ways of targeting this population.

### Glioblastoma stem cell enriched populations have more abnormal spindles following treatment with Aurora A inhibitors

Since GSCs display a more pronounced accumulation of γ-tubulin at the centrosome during mitotic entry, we reasoned that this could be an interesting point of attack for a targeted therapeutic approach. For this purpose we focused on AurA, which is a major regulator of mitotic centrosome maturation. The differences in centrosome dynamics could not be attributed to different levels of activity of AurA in the two cell populations (supplementary Fig. S2B–C and Fig. 2B). To analyse the requirement for AurA activity in the two populations, we treated stem and differentiated glioma cells with the AurA inhibitor MLN8237, using low dose levels that only had subtle effects on overall AurA kinase activity (supplementary Fig. S2C). Given that MLN8237 has a selectivity of more than 200-fold for AurA over Aurora kinase B (Manfredi et al., 2011), we can assume that any observed effects are AurA specific. Interestingly, treatment with the inhibitor induced more abnormal spindles in GSC enriched populations at all dose levels compared to more differentiated cells: 56% *vs.* 14% at 25 nM, 75% *vs.* 29% at 50 nM and 79% *vs.* 47% at 100 nM, respectively (Fig. 2C). The two populations of cells also exhibited a different response to AurA inhibition in terms of the type of spindle defect. GSC enriched populations showed a dramatic increase only in monopolar spindles, while their more differentiated counterparts showed a moderate increase in both monopolar and multipolar spindles (Fig. 2C). Fig. 2D shows representative images of treated cells. These data suggest that GSCs are highly susceptible to subtle changes in AurA activity.

### Aurora A inhibition induces an increase in polyploidy

**Figure 3:**
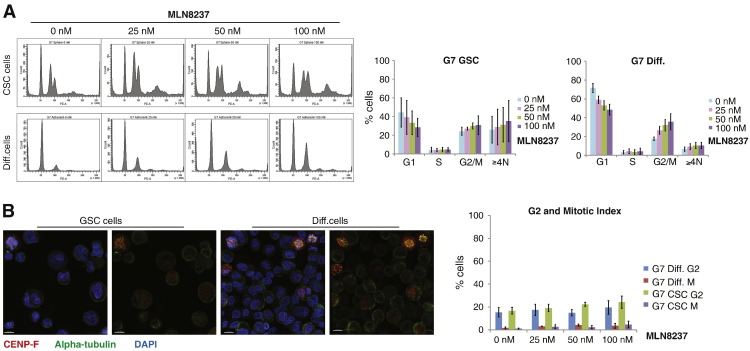
Aurora A inhibition does not cause a significant G2/M arrest in glioblastoma cells. (A) Cells were treated with MLN8237 (0, 25, 50 and 100 nM) and after 24 h they were fixed, stained with propidium iodide (PI) and analysed for DNA content: on the left are representative FACS diagrams of GSC and diff. cells; on the right, two diagrams show percentages of cells in the various phases of the cell cycle, quantified in the FACS analysis. (B) Cells were treated with MLN8237 (0, 25, 50 and 100 nM) and after 24 h they were fixed and stained for α tubulin (green), CENP-F (red) and DAPI (blue), to visualise G2/M cells: on the left are representative images of GSC and diff. cells; on the right, a diagram shows the percentages of cells in G2 and M in the two populations ± MLN8237. An average of 309 cells/condition/experiment were identified randomly and scored. All results are representative of three independent experiments. Error bars indicate means ± SD.

To further understand the consequences of AurA inhibitor treatment on GSCs we analysed parameters of cell cycle distribution in the two cell populations. Several studies have reported a G2/M arrest following inhibition of AurA, either by small molecule inhibitors or by RNAi (Gorgun et al., 2010). In our study the baseline cell cycle profiles of the two populations differed significantly: GSC enriched populations had a higher percentage of cells with 4 N and > 4 N DNA content (Fig. 3A). Cells with a 4 N FACS profile can be in G2, M or a quatroploid G1 phase. To distinguish between these cell cycle states, we scored the percentage of cells in G2 and M by immunofluorescence using CENP-F, α-tubulin and DAPI staining (for a representative example, see Fig. 3B). The G2/M fraction was similar in the two populations, confirming that the difference in cells with 4 N DNA content was due to polyploidy. Cell cycle profiles of the two populations 24 h after treatment with MLN8237 showed an increase in the 4 N and > 4 N DNA content fraction in both populations. Immunofluorescence analysis showed only subtle increases in the percentage of G2 and M phase cells after treatment, suggesting that AurA inhibition does not induce a prolonged G2/M arrest in these cells, despite a significant increase of mitotic aberrations following MLN8237 treatment (Fig. 2).

**Figure 4:**
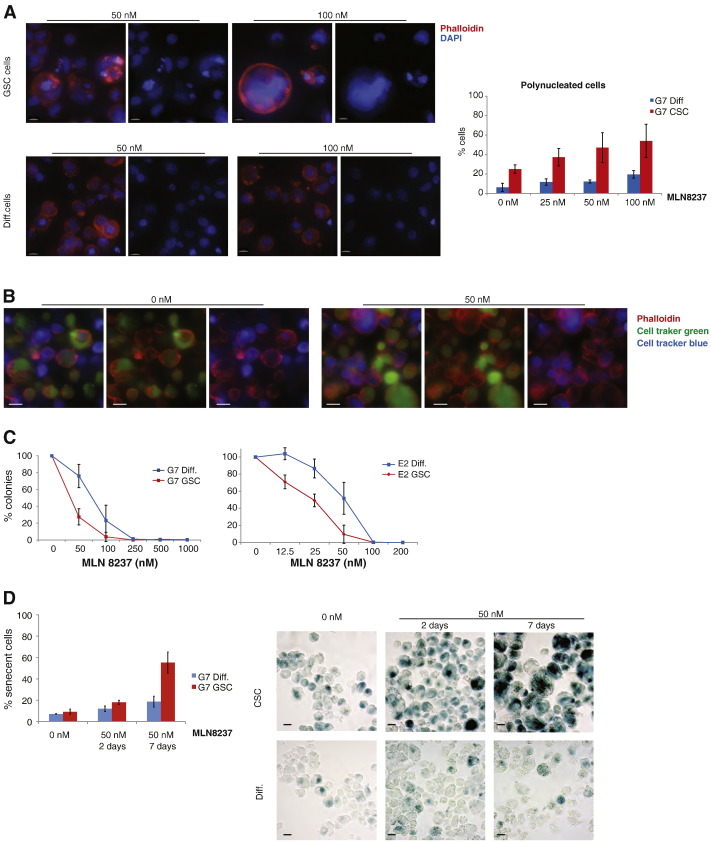
Glioblastoma stem cells are killed more efficiently by MLN8237. (A) Cells were treated with MLN8237 (0, 25, 50 and 100 nM) and after 24 h they were fixed, stained with phalloidin (red) and DAPI (blue), to visualise the cell cortex and nucleus: on the left are representative images of GSC and diff. cells (scale bar 10 μm); on the right, a diagram shows the percentages of polynucleated cells in the two populations ± MLN8237. An average of 221 cells/condition/experiment were identified randomly and scored. (B) Cells were incubated separately with CellTracker Green and Blue, re-suspended in fresh medium, incubated for 24 h ± 50 nM MLN8237 and then fixed and stained with phalloidin: representative images of sphere cells ± MLN8237 (scale bar 10 μm). (C) Clonogenic assays showing survival of sphere and adherent cells ± MLN8237. (D) Cells were treated with MLN8237 (0 and 50 nM) and after 2 or 7 days they were fixed and stained for β-galactosidase: on the left, a diagram shows the percentages of senescent cells in the two populations ± MLN8237; on the right are representative images of sphere and adherent cells ± MLN8237. (scale bar 10 μm). An average of 233 cells/condition/experiment were identified randomly and scored. All results are representative of three independent experiments. Error bars indicate means ± SD.

To confirm and characterise the moderate increase in the ≥ 4 N fraction, we stained with phalloidin cells after AurA inhibition. This analysis detected an increasing number of giant polynucleated cells that was more pronounced in the GSC population: 37%, 47% and 54% of these cells were polyploid after 25, 50 and 100 nM treatments, compared with 12%, 12% and 20% in the more differentiated populations (Fig. 4A). That these percentages were higher than indicated by the FACS data is probably explained by the large size of some polyploid cells causing them to evade FACS analysis.

Polyploidy can be caused by several mechanisms: abnormal mitosis, endoreduplication, cell fusion and entosis (Krajcovic and Overholtzer, 2012). Given that cell appearance does not distinguish between the products of cell fusion and mitotic failure, and that entosis has been observed mainly under non-adherent growth conditions (Overholtzer et al., 2007), we tested the extent to which these different processes were responsible for baseline and post-treatment levels of polyploidy amongst GSCs. Cells were marked separately with cell tracking dyes, mixed then incubated for 24 h with or without MLN8237 (Fig. 4B). In both treated and untreated samples the vast majority of polyploid cells originated from the same mother cell, suggesting that in GSCs polyploidy is not a result of cell fusion or entosis, but rather a consequence of mitotic defects.

### Increase in polyploidy correlates with increased sensitivity to Aurora A inhibition and induction of senescence

Our analysis suggests that GSCs are prone to undergoing mitotic failure and are highly susceptible to subtle changes in AurA activity levels. The observed increase in monopolar spindles does not cause a prolonged mitotic arrest suggesting that these cells are prone to mitotic slippage. This is also reflected in the rapid polyploidization observed after MLN8237 treatment. Taken together these observations indicate that chemical inhibitors of mitosis might be useful therapeutic agents that specifically target the GSC population. We tested this hypothesis by measuring the sensitivity of glioblastoma stem cell enriched and more differentiated populations to MLN8237 and found that two independent GSC lines were indeed killed more efficiently by the AurA inhibitor (Fig. 4C). We further tested our hypothesis by analysing the effect on clonogenicity of inhibiting another centrosome kinase, Plk1, with BI2536: again, two independent GSC lines had a lower survival than their differentiated counterparts (supplementary Fig. 3).

In order to understand the cause of death in glioblastoma stem cell enriched and more differentiated populations following AurA inhibition, we measured levels of apoptosis and senescence. While MLN8237 did not increase apoptosis as judged by cleaved Caspase 3 levels in either population (supplementary Fig. 4A), a significant increase in the number of senescent cells was observed. Seven days after AurA inhibition, 55% of GSCs expressed a marker of senescence, compared with only 19% of differentiated cells (Fig. 4D). The negligible level of apoptosis is consistent with some published studies (Huck et al., 2010; Liu et al., 2013) but not with others (Gorgun et al., 2010). Recent literature is also conflicting with regard to the correlation between cell fate following AurA inhibition and p53 status (Liu et al., 2013; Nair et al., 2012; Liu et al., 2013). To test whether the different response to MLN8237 was due to p53 status, we analysed levels of p53 expression in glioblastoma stem cell enriched and more differentiated populations in three primary cell lines: there was no common pattern of p53 levels in the various cell lines when comparing the two subpopulations (supplementary Fig. 4B–D). This suggests that the increased sensitivity of GSCs to AurA inhibition is not dependent on p53 status.

Several studies in a variety of cancer models have shown that cellular senescence is induced *in vivo* by chemotherapy and radiotherapy (Roninson, 2003). Although a large body of evidence links senescence to tumour suppression, recent data suggests that, in a minority of cancer cells, senescence associated polyploidy can be reversible and might constitute a survival mechanism. A clinicopathological analysis of specimens from patients with non-small cell lung cancer undergoing surgery after neo-adjuvant chemotherapy showed that β-galactosidase staining was correlated with decreased overall survival (Wang et al., 2013). Moreover, one of the features of senescent cells is the acquisition of a secretory phenotype, which creates a niche that can affect adjacent cells (Rodier and Campisi, 2011). Amongst the released factors is IL-6 (Coppe et al., 2008), which has been reported to promote GSC survival and tumour growth (Wang et al., 2009). These findings suggest a possible link between chemotherapy-induced senescence, GSCs and treatment resistance. Our survival data clearly indicate that senescence following MLN8237-induced mitotic failure causes a reduction of neurosphere formation in GSCs and generally decreases the clonogenic potential of glioma cells. Hence we propose that induction of senescence by polyploidy could be a promising anticancer strategy that targets GSCs, rather than a survival mechanism. Given the limitations of a single cell survival assay in this context, our findings highlight the need for *in vivo* studies and pathological analysis to clarify the role of senescence associated polyploidy in GSC biology and treatment outcomes.

Another significant outcome of our study is the difference in centrosome maturation and mitotic spindle phenotypes between GSC enriched and differentiated populations. To our knowledge there are no previous reports on this aspect of GSC biology. The high susceptibility of GSCs to subtle changes in levels of kinases involved in the centrosome cycle is particularly interesting if we consider the literature on the role of symmetric and asymmetric divisions in cancer. Defects in regulation of switch between asymmetric and symmetric divisions have been speculated to be involved in carcinogenesis (Morrison and Kimble, 2006), and therefore might be strongly linked to generation of GSCs. GSCs *in vitro* divide mainly by symmetric division, but are able to increase the asymmetric mode following growth factor withdrawal, *i.e.* a differentiation stimulus (Lathia et al., 2011). Normal adult stem cells seem to switch from asymmetrical to symmetrical division following injury (Morrison and Kimble, 2006). The study mentioned previously (Chen et al., 2012), which used a genetically engineered mouse model of glioma, reported data on transient subsets of highly proliferating tumour cells post-chemotherapy. In this study the growth patterns were consistent with an initial prevalence of symmetric divisions followed by a switch to asymmetrical mode. Based on this data and on our findings, we speculate that GSC mitosis confers more plasticity and increased regenerative ability to these cells, but also renders them more susceptible to mitotic failure.

Mechanisms regulating mitosis, as well as senescence, in GSCs, are still poorly understood and need to be investigated further, especially with pathology studies that would be able to confirm whether our *in vitro* findings apply to GSCs in their natural microenvironment.

## Materials and methods

### Cell lines and cell culture

E2 and G7 primary glioblastoma cell lines were derived from freshly resected GBM specimens as previously described (Fael Al-Mayhani et al., 2009) and generously provided by Colin Watts (Cambridge). Tissue collection protocols were compliant with the UK Human Tissue Act 2004 (HTA Licence ref 12315) and approved by the local regional Ethics Committee (LREC ref 04/Q0108/60). Informed consent was obtained from each patient before surgery. Briefly, anonymised patient resection specimens were homogenised and seeded in serum free (SF) media to form spheroid aggregates which were then collected and plated onto extracellular matrix coated flasks. (ECM 1:10 dilution, Sigma). Cells were allowed to form a primary monolayer then passaged in SF medium. Each cell line was subsequently cultured as paired cancer stem cell enriched (GSC) and differentiated (diff) cell lines by passaging in either SF media or differentiating media (DM). GSC enriched populations were cultured as neurospheres in Neurobasal-A medium (Invitrogen) supplemented with B-27 (Invitrogen), epidermal growth factor (20 ng/ml, Invitrogen PHG0313), fibroblast growth factor (20 ng/ml, Invitrogen PHG0263), glutamine and penicillin/streptomycin (referred to as ‘sphere cells’ or ‘GSC’ in figures). Differentiated populations were derived from these cells by culturing them as adherent cells in MEM supplemented with HyClone (Thermo Scientific HyClone, 12822966), NEAA, glutamine and penicillin/streptomycin (referred to as ‘adherent’ or ‘diff’ cells in figures).

### Reagents

In all experiments with AurA inhibition we incubated cells with MLN8237 (Millenium) for 24 h. For clonogenic survival assays we added the reagents to the plates and left them for the whole duration of the experiment.

### Immunostaining

For centrosome maturation, mitotic spindle and cell cycle analysis, cells were collected from flasks, spun down, re-suspended, pipetted on Concanavalin A (Sigma) coated coverslips and fixed with 70% methanol for 5 min. For P-AurA analysis, cells were cytospun on Concanavalin A coated coverslips and fixed with 4% paraformaldehyde and 70% methanol, sequentially for 5 min each. Cells were permeabilized in PBS 0.3% Triton for 5 min, blocked in 3% BSA for 30 min and probed with primary antibodies for 60 min. Slides were rinsed and probed with Alexa Fluor secondary antibodies (Invitrogen) for 60 min. Coverslips were mounted using ProLong Gold mounting solution containing DAPI (Molecular Probes). The following primary antibodies were used: HLA Class 1ABC (EMR8-5, Abcam ab70328), Ki67, CD133, nestin, Sox2, GFAP, α tubulin (Abcam ab7291, ab18251), γ tubulin (Abcam ab11316), centrin-2 (gift from Elmar Schiebel), CENP-F (Abcam ab5), and P-AurA (Cell Signalling 3079).

Images were acquired on a microscope (DeltaVision) equipped with a UPLS Apochromat NA 1.40, 60 × or 100 × oil immersion objective (Olympus), standard filter sets (excitation 360/40, 490/20, and 555/28; emission 457/50, 528/38, and 617/40), and a camera (CoolSNAP HQ2; Photometrics). Z series of 0.3 μm stacks were acquired using softWoRx software (version 4.0.0; Applied Precision) and deconvolution was performed using SVI Huygens Professional Deconvolution Software (Version 3.5). For quantitative data on γ tubulin and P-AurA centrosomal localization, DeltaVision files were imported into Imaris software (version 6.3.0; Bitplane) for 3D rendering measurements using the surface rendering algorithm for γ tubulin and P-AurA signals. Measurements were then exported to Excel (Microsoft) and plotted. The data was further analysed using Mann–Whitney *U* test.

For ploidy analysis, cells were collected from flasks, spun down, re-suspended, pipetted on Concanavalin A coated coverslips and fixed with 4% paraformaldehyde for 10 min. Cells were permeabilized, blocked and probed with phalloidin (Invitrogen) for 30 min. Coverslips were mounted as above.

### FACS analysis

It was performed as previously described (Hegarat et al., 2011).

### Cell tracking

Cells were incubated separately with 1 μM CellTracker Green CMFDA (5-Chloromethylfluorescein Diacetate) and 1 μM CellTracker Blue CMAC (7-Amino-4-Chloromethylcoumarin) (both from Molecular Probes) for 45 min, spun down, re-suspended in fresh medium and incubated for 24 h ± 50 nM MLN8237. Cells were then fixed and stained with phalloidin as in the ploidy analysis. Coverslips were mounted using Fluoromount (Sigma).

### Clonogenic survival assay

Clonogenics were performed plating the cell suspension in 96 well plates, at an ideal concentration of 1 cell/well (200 μl). Each subpopulation of cells was plated in the appropriate medium. EGF and FGF are added to the GSC plates on days 5, 10 and 15. Neurospheres and adherent colonies were counted on day 21 using the Gel Count (Oxford Optronix) and methylene blue staining, respectively.

### β-galactosidase staining

Cells were treated with MLN8237 and after 2 and 7 days they were fixed and stained for β-galactosidase according to the manufacture's protocol (Abcam). Images were acquired on a microscope Axio Lb A1 (Zeiss) equipped with an AxioCam ERc 5 s and a 40 × objective.
