# Supplementary material for: Differential sensitivity of Glioma stem cells to Aurora kinase A inhibitors: Implications for stem cell mitosis and centrosome dynamics
Source: Stem Cell Res. 2014 Jul;13(1):135–43. doi: 10.1016/j.scr.2014.05.001 (PMC4085484; doi:10.1016/j.scr.2014.05.001)

Supplemental Figure 1. (A) Cells ($5x10^5$) cultured in serum-free conditions (GSC) or in the presence of serum (tumour bulk/diff) were dissociated with Accutase and analysed for CD133 and GFAP expression by FACS. Diagrams show the average (error bars= SEM) of three independent experiments where percentage of either CD133 or GFAP-expressing cells were quantified **(B)** Immunofluorescence microscopy for the stem cell marker nestin and the astrocytic differentiation marker GFAP was performed in fixed cells cultured as monolayers in serum-free conditions (on Matrigel-coated coverslips) or in serum-containing differentiated conditions. **(C)** Analysis of E2 tumour load in mouse brain sections was performed using ZenBlue Analysis software. Number of Ki67-expressing cells was quantified from full brain images (bottom panel) by selecting Ki67+ cells. Upper panel shows IHC for Ki67+ cells (yellow dots) whereas middle panel presents cells detected by software after selection process has been set to recognise Ki67+ nuclei.

Supplementary Figure 2 (A) γ tubulin staining is specifically localized at centrosomes. Cells were stained for centrin-2 (green), γ tubulin (red) and DAPI (blue), to confirm specificity of γ tubulin staining at centrosomes. The following primary antibodies and reagents were used: γ tubulin (Abcam ab11316), centrin-2 (gift from Elmar Schiebel) and ProLong Gold mounting solution containing DAPI (Molecular Probes). Acquisition of images as in Figure 1. **(B)** Stem cell enriched and differentiated populations do not differ in total and phosphorylated Aurora A expression, baseline and after MLN8237 treatment. **(A)** and **(C)** Total Aurora A expression in stem cell enriched and differentiated populations of three different primary cell lines (E2, G7 and S2). **(C)** Total and phosphorylated Aurora A expression, baseline and after 24 hours of MLN8237 treatment; "Control" is a sample of HeLa cells synchronized in M phase with 100 ng/uL nocodazole for 16-18h. For cell lysate preparation, cells were washed once in PBS and lysed in 40 µl IP lysis buffer (20mM Tris pH 7.5, 137mM NaCl, 10% glycerol, 1% triton, 2mM EDTA, 0.05% b-mercaptoethanol, protease and phosphatase inhibitors) and 10 µl 5x sample buffer (0.01% bromophenol blue, 62.5mM Tris-HCl pH 6.8, 7% SDS, 20% sucrose and β-mercaptoethanol). Cell lysates were incubated on ice for 20 min, sonicated, boiled for 5 minutes at 95°C and loaded to electrophoresis in SDS-12% polyacrylamide gel. This was followed by semi-dry transfer to a nitrocellulose membrane, which was then incubated overnight at 4°C with the following antibodies: anti-Aurora A (Epitomics 1800-1), anti-phosphorylated Aurora A (Cell Signaling 3079) and anti-histone H3 (Millipore, 05-499).

Supplementary Figure 3. Stem cell enriched and differentiated populations do not show an increase in apoptosis following MLN827 treatment and their fate is not correlated to p53 status. **(A)** Cleaved Caspase 3 levels in GSC and differentiated populations baseline and after 24 hours of MLN8237 treatment. **(B-C)** Total p53 expression baseline and after 24 hours of MLN8237 treatment in stem cell enriched and differentiated populations of two primary cell lines (G7 and E2). **(D)** Baseline total p53 expression in stem cell enriched and differentiated populations (S2).

Supplementary figure 4. Glioblastoma stem cells are killed more efficiently by BI2536, a Plk1 inhibitor. Clonogenic assays showing survival of GSC and diff. cells of two different cell lines (E2 and G7) ± BI2536. Clonogenics were performed as in figure 4. All results are representative of three independent experiments. Error bars indicate means ± SD.

Supplementary Figure 1

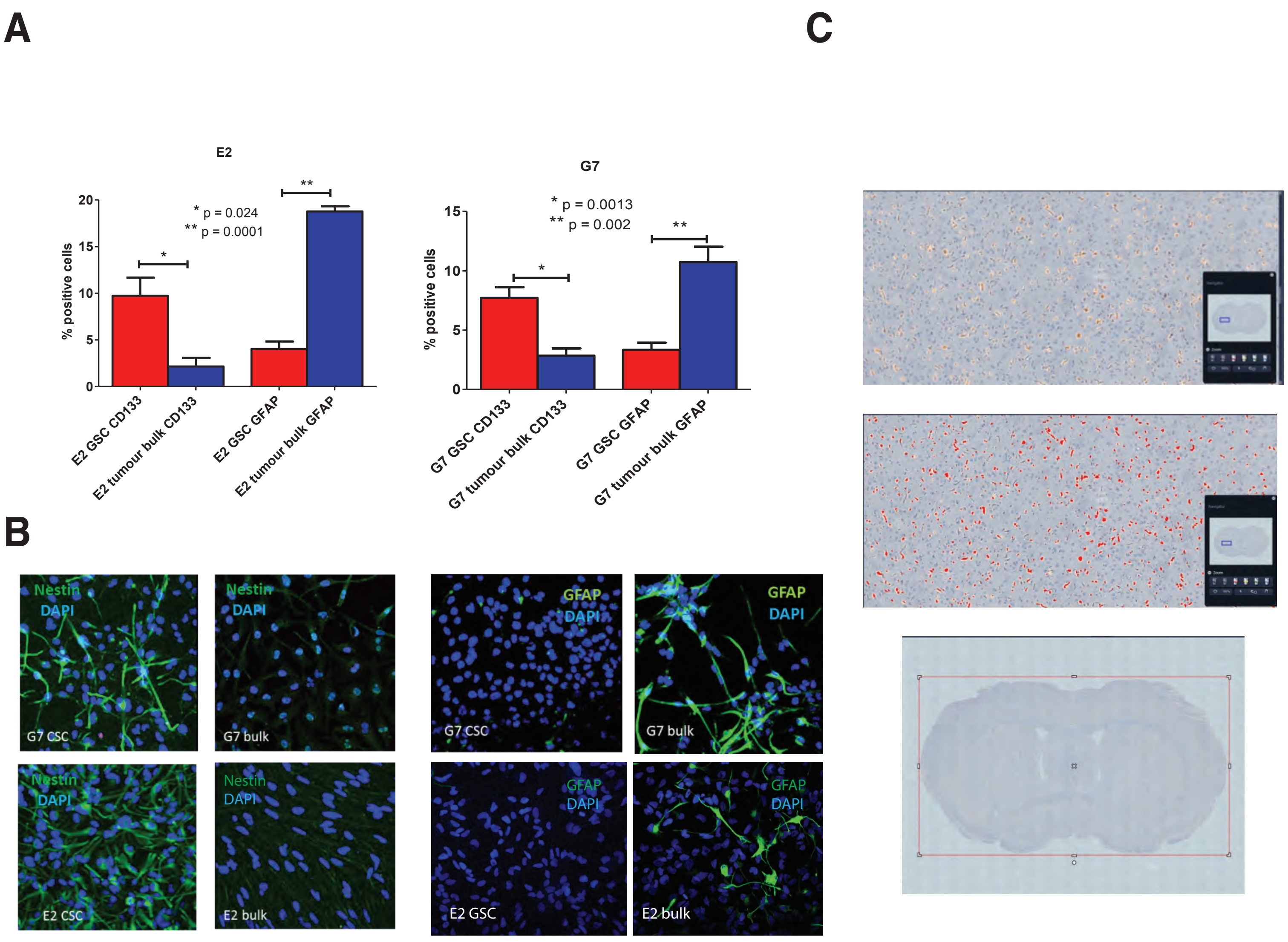

Supplementary Figure 2

A

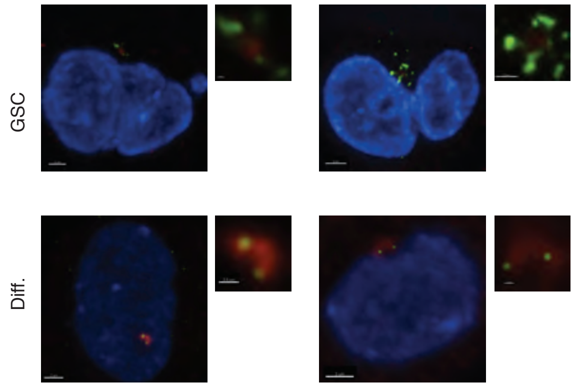

B

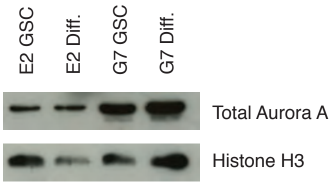

C

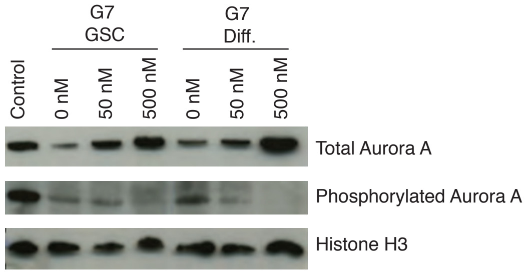

D

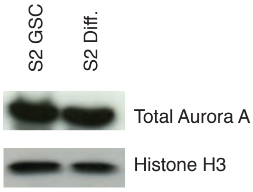

Supplementary Figure 3

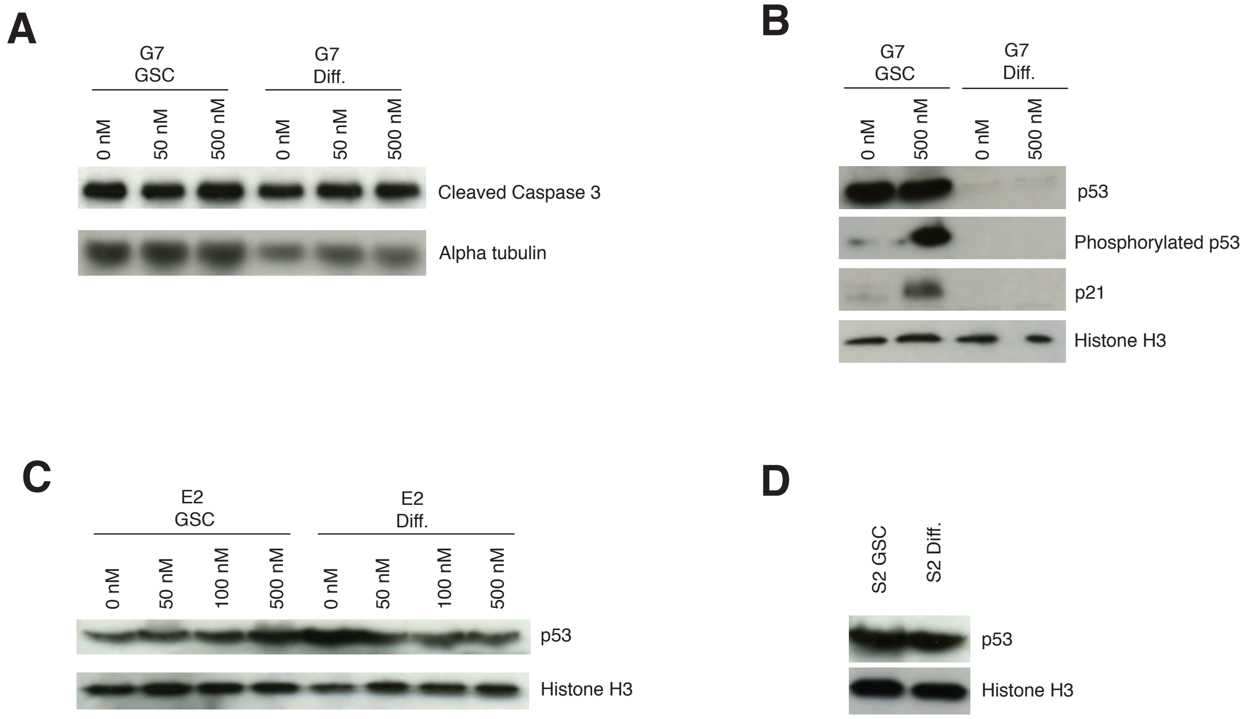

Supplementary Figure 4

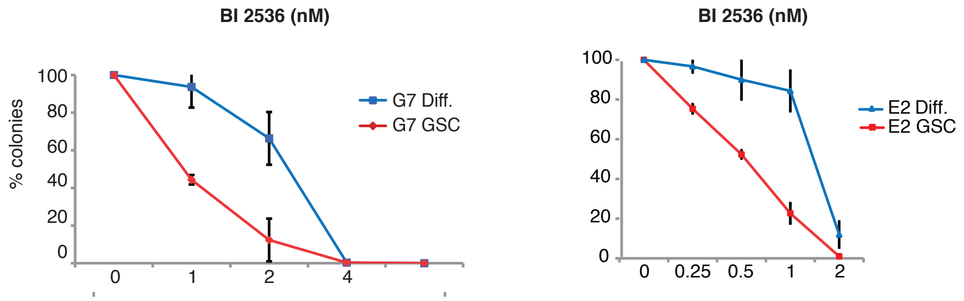

Supplement: Supplementary file 1 — Supplementary figures. [file mmc1.pdf]
